# Complement C1q Binding Protein (C1QBP): Physiological Functions, Mutation-Associated Mitochondrial Cardiomyopathy and Current Disease Models

**DOI:** 10.3389/fcvm.2022.843853

**Published:** 2022-03-02

**Authors:** Jie Wang, Christopher L-H Huang, Yanmin Zhang

**Affiliations:** ^1^National Regional Children's Medical Center (Northwest), Xi'an, China; ^2^Key Laboratory of Precision Medicine to Pediatric Diseases of Shaanxi Province, Xi'an, China; ^3^Shaanxi Institute for Pediatric Diseases, Xi'an, China; ^4^Xi'an Key Laboratory of Children's Health and Diseases, Xi'an, China; ^5^Physiological Laboratory, University of Cambridge, Cambridge, United Kingdom; ^6^Department of Cardiology of Xi'an Children's Hospital, Affiliated Children's Hospital of Xi'an Jiaotong University, Xi'an, China

**Keywords:** C1QPB, mutation, combined oxidative phosphorylation deficiency, mitochondrial cardiomyopathies, physiological functions, disease models

## Abstract

Complement C1q binding protein (C1QBP, p32) is primarily localized in mitochondrial matrix and associated with mitochondrial oxidative phosphorylative function. C1QBP deficiency presents as a mitochondrial disorder involving multiple organ systems. Recently, disease associated C1QBP mutations have been identified in patients with a combined oxidative phosphorylation deficiency taking an autosomal recessive inherited pattern. The clinical spectrum ranges from intrauterine growth restriction to childhood (cardio) myopathy and late-onset progressive external ophthalmoplegia. This review summarizes the physiological functions of C1QBP, its mutation-associated mitochondrial cardiomyopathy shown in the reported available patients and current experimental disease platforms modeling these conditions.

## Introduction

Mitochondrial diseases form a diverse group of heritable disorders caused by a wide spectrum of mutations in nuclear or mitochondrial genes (1, 2). The nuclear DNA encodes over 1,000 mitochondrially localizing proteins, translated in the cytoplasm, and translocated to the mitochondria. The mitochondrial (mt-)DNA encodes 37 genes including 22 tRNAs and two rRNAs. The mt-DNA is essential for mt-DNA-specific translation of the 13 encoded respiratory chain subunits (3). Dysfunction in mitochondrial respiratory chain function and cellular energy production affects different tissues, owing to critical dependence of the heart on oxidative metabolism; cardiac involvement in mitochondrial disease is common and may occur as the principal clinical manifestation or part of multisystem disease (4, 5). Cardiovascular involvement in mitochondrial disease resulted in lower survival rates to age 16 years than in patients without heart disease (6).

*C1QBP* comprises 6 exons located on the short arm of chromosome 17p13.2. It is highly conserved through evolution (7): the human and rat/mouse cDNA sequences are almost identical (8, 9). *C1QBP* encodes the complement component 1Q subcomponent-binding protein (C1QBP, p32). This widely expressed multifunctional protein is predominantly localized in mitochondrial matrix (10, 11). Its N-terminal mitochondrial targeting peptide is proteolytically processed after it reaches the mitochondrial matrix. Here it forms a doughnut-shaped homotrimeric complex (12–14).

C1QBP is reported to exert pleiotropic effects on many cellular processes, including mitochondrial homeostasis, mitochondrial oxidative phosphorylation (OXPHOS) and in nucleus–mitochondrial interactions, inflammation, and cancer (15–19). Biallelic *C1QBP* mutations were recently identified manifesting as combined oxidative phosphorylation deficiency (COXPD) in an autosomal recessive inherited pattern. This involved multiple systems including heart, liver, skeletal muscle, eye and nervous system (20–24). However, cardiomyopathy, whose exact underlying mechanisms remain elusive, is the major phenotype. This review summarizes physiological functions of C1QBP, its mutation-associated mitochondrial cardiomyopathy, and current disease models.

## Physiological Functions of C1QBP

C1QBP is highly expressed in cells with substantial energy metabolism such as cardiac and skeletal muscle (25). It can exist in numerous cellular compartments but is predominantly targeted to the mitochondria reflecting the mitochondrial targeting sequence in its 73N-terminal amino acids. The C1QBP crystal structure reveals three monomers forming a doughnut-shaped quaternary structure with an unusually asymmetric surface charge distribution. It shows a fold comprising seven consecutive antiparallel β-strands flanked by one N-terminal and two C-terminal α-helices (25–29).

Mitochondrial C1QBP is essential for OXPHOS. It pivotally supports translation of the mitochondrially encoded respiratory chain protein complexes I, III, IV and V (30). C1QBP knockdown decreased complexes I, III, IV, and V but not complex II levels, reducing complex I and IV enzymatic activity (16). Chen et al. reported that C1QBP binds to the core, dihydrolipoyl-lysine-residue acetyltransferase (DLAT), component of the matrix multienzyme pyruvate dehydrogenase (PDH) complex (31). PDH is important in energy homeostasis, linking glycolysis and the tricarboxylic acid (TCA) cycle. PDH catalyzes pyruvate conversion to acetyl-CoA used in the TCA cycle to generate ATP (32). C1QBP regulates OXPHOS through binding to DLAT, providing a novel molecular mechanism by which C1QBP regulates cellular respiration (27).

C1QBP is required for development and maintenance of normal cardiac function (15). Cardiomyocyte specific C1QBP deletion resulted in contractile dysfunction, cardiac dilatation and fibrosis. C1QBP deficiency decreases COX1 expression and oxygen consumption rates, increased oxidative stress, further leading to cardiomyocyte dysfunction. In cardiomyocytes, C1QBP acts as an RNA and protein chaperone modulating mitochondrial translation and function (15). In addition, 5' adenosine monophosphate-activated protein kinase A (AMPKA) was constitutively phosphorylated, and eukaryotic translation initiation factor 4E (eIF4E)-binding protein 1 (4E-BP1) and ribosomal S6 kinase (S6K) were less phosphorylated in C1QBP deficient myocytes, suggesting impaired rapamycin signaling. Metabolic analysis also demonstrated an impaired urea cycle in C1QBP deficient hearts (15).

Mitochondrial morphology is closely linked to energy metabolism. Reduced OXPHOS and enhanced glycolysis correlates with mitochondria fragmentation and mitochondrial matrix expansion (33). C1QBP is required to maintain normal mitochondrial structure and is critical in protecting mitochondria from fragmentation and swelling by inhibiting OMA1-dependent proteolytic processing of the optic atrophy type 1 GTPase protein (OPA1) (15, 28). Cardiac mitochondria from C1QBP-deficient mice showed disordered alignment, enlargement and abnormalities in their internal structure (15). Furthermore, the mitochondrial network was fragmented rather than fibrillar when C1QBP was not expressed (16).

The crystal structure of C1QBP has been determined. This is compatible with an association with regulation of mitochondrial Ca^2+^. It can form a pore-like homotrimer that could serve as a high-capacity divalent cation storage protein. C1QBP contains 282 amino acid residues. Twenty three percent of these residues are glutamic and aspartic acids distributed on the trimer surface. This characteristic acidic surface is reminiscent of the major sarcoplasmic reticular Ca^2+^ storage protein calsequestrin (34). The latter modulates intracellular Ca^2+^ concentration and affects membrane Ca^2+^ transport rates into sarcoplasmic reticular vesicles. C1QBP may similarly modulate Ca^2+^ levels in the mitochondrial matrix (14). Xiao et al. proposed that C1QBP protein is additionally a positive regulator of mitochondrial Ca^2+^ uptake (35). Koo et al. and Choi et al. similarly confirmed that mitochondrial C1QBP protein has a regulatory effect on mitochondrial Ca^2+^ uptake (36–38). Oxidative metabolism strongly varies with mitochondrial Ca^2+^ levels (39). PDH, NAD-isocitrate dehydrogenase and oxoglutarate dehydrogenase are all regulated by intramitochondrial Ca^2+^ levels either directly or indirectly (40). C1QBP may thereby regulate mitochondrial OXPHOS by modulating Ca^2+^ concentrations (14).

C1QBP expression increases in multiple cancer cells in human, including breast, endometrial, ovarian, prostate, melanoma, lung, and colon cancer (41–49). C1QBP may be pivotal in tumor cell survival, growth and metastatic invasion through interacting with critical molecules, including those of the complement and kinin systems, in the tumor cell microenvironment (25). C1QBP may be needed to sustain tumor cell growth by maintaining respiration and OXPHOS. C1QBP knockdown tumor cell lines showed decreased complex I, III, IV, and V subunit levels (50). Zhang et al. (51) suggested that C1QBP further regulated protein kinase Cζ activity and modulated EGF-induced cancer cell chemotaxis. It was additionally identified as a novel regulator of cancer metastasis that may serve as a target for breast cancer therapy (50).

Finally C1QBP appears critical in inflammation processes and responses to infection (25). It binds to a wide variety of plasma and cell surface, and pathogenic microorganism proteins. It is critical in modulating fibrin formation, particularly at local sites of immune injury and/or inflammation and activating the kinin/kallikrein system. It is also able to generate the powerful vasoactive peptide bradykinin largely responsible for the swelling seen in angioedema (17–19).

## C1QBP Mutations and Human Mitochondrial Cardiomyopathy

Biallelic C1QBP mutations were first reported in four individuals by Feichtinger et al. (20). Biallelic C1QBP mutation caused a COXPD 33 (OMIM:617713). In the reported 12 cases with C1QBP mutations, phenotypes were typically severe, even fatal (20–24). They present at any age and cover a wide spectrum of clinical manifestations including intrauterine growth restriction, cardiorespiratory arrest, cardiac hypertrophy, cardiac failure, ventricular arrhythmias, hepatomegaly, exercise intolerance, progressive external ophthalmoplegia (PEO), cerebral hemorrhages/edema and nervous system dysfunction.

### Mutations in C1QBP and Clinical Characteristics

**Figure 1:**
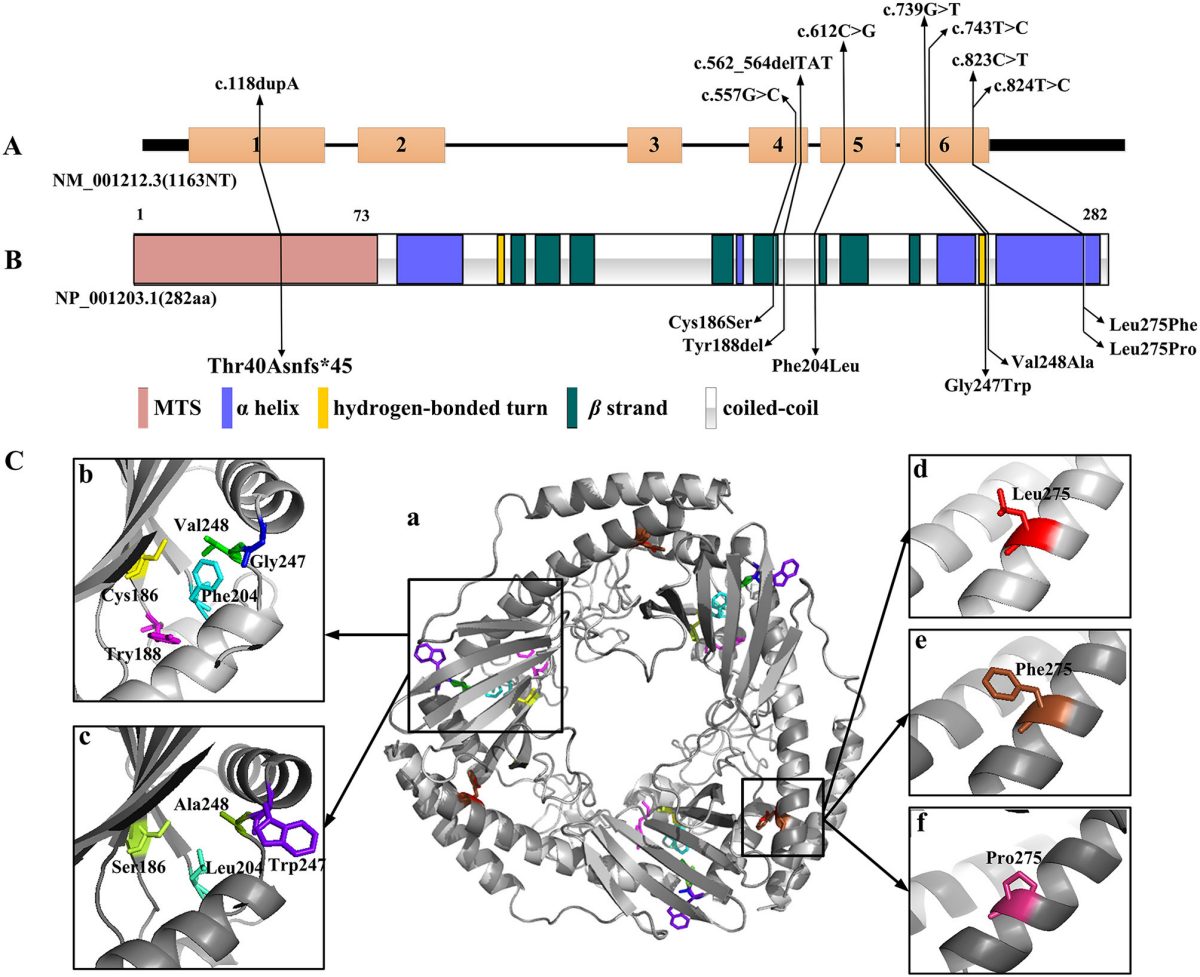
C1QBP variants in gene and protein structure. **(A)** Gene structure with exons and introns showing the localization of the variants. **(B)** Secondary structure of the C1QBP indicating the positions of the variants. MTS indicates the mitochondrial target sequence. **(C)** Inspection of the C1QBP structure performed using PyMOL (PDB accession codes 1P32, https://www.rcsb.org/structure/1P32). a: Predicted three-dimensional structure of the C1QBP protein; b and d: wild type; c, e and f: mutation type. Residue changes are colored in the structure: Cys186, yellow; Ser186, lemon; Tyr188, magenta; Phe204, cyan; Leu204, green-cyan; Gly247, blue; Trp247, purple-blue; Val248, green; Ala248, forest; Leu275, red; Phe275, brown; Pro275, warm pink.

Amongst the 12 reported patients there were eight C1QBP amino acid changes, summarized in Table 1. Figure 1 illustrates the gene (Figure 1A) and protein structures (Figures 1B,C) indicating the positions of C1QBP variants. Table 2 summarizes the relative frequency of symptoms associated with biallelic variants in C1QBP and the relates the Human Phenotype Ontology (HPO) terms in which C1QBP mutatins should be suspected when a patient presents with cardiomyopathy, especially left ventricular hypertrophy, cardiomegaly, exercise tolerance, ptosis and PEO.

**Table 1:** Disease-associated mutations of C1QBP in mitochondrial disease.

**Case No**.	**Site of mutation**	**Type of mutation**	**Location**		**mtDNA damage**	**MRC complex activities**	**Ethnicity**	**Gender**	**Age of onset**	**Outcome**	**Involved system**	**Symptoms**		**References**
			**exons**	**protein**								**Cadiovasular system**	**Other system**	
1	c. 557G>C; p. Cys186Ser; c. 612C>G; p. Phe204Leu	compound heterozygous mutations	4; 5	β strand; coiled- coil region	Copy number variation	Muscle: I/CS: 27% II/CS: 64% III/CS: 8% IV/CS: 82%	British	male	4 days	deceased (18 days)	heart; CNS; kidney	cardiorespiratory arrest, asymmetric left ventricular cardiomegaly	multiple cortical, ventricular, and subdural hemorrhages and cerebral edema, burst suppression-like electrical discharges, subclinical seizures, congenital nephrosis, hypothyroidism, disseminated intravascular coagulopathy	Feichtinger et al. (20)
2	c.739G>T; p. Gly247Trp; c.824T>C p. Leu275Pro	compound heterozygous mutations	6	hydrogen-bonded turn; αC helix	Copy number variation	Liver: I/CS: 6% II/CS: 36% III/CS: 22% IV/CS: 13%	Japanese	female	birth	deceased (4 days)	heart; liver	cardiomegaly	hepatomegaly	Feichtinger et al. (20)
3	c. 823C>T; p. Leu275Phe	homozygous mutations	6	αC helix	mtDNA deletions	Muscle: I/CS: 12% I+III/CS: 63% III/CS: 8% IV/CS: 6%	Austrian	male	5 years	alive (22 years)	heart; liver; PNS; muscle; eye	left ventricular hypertrophy	increased transaminases; sensory peripheral neuropathy, exercise intolerance with fatigue and vomiting, astigmatism, amblyopia, ptosis, PEO	Feichtinger et al. (20)
4	c.562_564del; p. Tyr188del	homozygous mutations	4	coiled- coil region	mtDNA deletions	Muscle: I/CS: 55% I+III/CS: 52% II/CS: 57% II+III/CS: 33% IV/CS: 46%	Italian	male	57 years	deceased (70 years)	heart; PNS; muscle; eye	left ventricular hypertrophy	diffuse neurogenic abnormalities and focal myogenic in the gluteus maximus, exercise intolerance, weakness, ptosis, PEO, post-traumatic depression, diabetes, sensorineural hearing loss	Feichtinger et al. (20)
5	c.612C>G p.Phe204Leu	homozygous mutations	5	coiled- coil region	mtDNA deletions	Muscle: I/CS: about 40% II/CS: about 140% III/CS: about 60% IV/CS: about 40%	Italian	female	28 years	alive (54 years)	eye; muscle;	Nil	PEO, bilateral ptosis, almost complete ophthalmoparesis, severe dysphagia, and rhinolalia	Marchet et al. (21)
6	c.562_564del p.Tyr188del	homozygous mutations	4	coiled- coil region	mtDNA deletions	Muscle: I/CS: about 80% II/CS: about 180% III/CS: about 100% IV/CS: about 100%	Italian	female	30 years	alive (65 years)	eye; muscle; PNS;	Nil	PEO; bilateral ptosis, hyposthenia, swallowing dysfunction, decreased exercise tolerance dysfunctions in executive and visuospatial areas	Marchet et al. (21)
7	c. 823C>T p. Leu275Phe	homozygous mutations	6	αC helix	Nil	NA	Chinese	male	1.5 years	alive (14 years)	heart; muscle; eye	left ventricular hypertrophy	decreased exercise tolerance; ptosis	Wang et al. (22)
8	c. 823C>T p. Leu275Phe	homozygous mutations	6	αC helix	Nil	NA	Chinese	male	2 years	alive (9 years)	heart; muscle; eye	left ventricular hypertrophy	decreased exercise tolerance; ptosis	Wang et al. (22)
9	c.743T>C p.Val248Ala	homozygous mutations	6	hydrogen-bonded turn	NA	NA	Syrian	male	fetuses	deceased (33 weeks gestational age)	heart; liver;	cardiomyopathy	IUGR, oligo/anhydramnios, generalized edema, cardio/hepatomegaly, cortical hemorrhages, and preterm birth	Alstrup et al. (23)
10	c.743T>C p.Val248Ala	homozygous mutations	6	hydrogen-bonded turn	NA	Fibroblasts: II: 0.57 (reference range, 0.38–0.76) III: 1.6 (reference range, 1.0–1.8) IV: 0.7 (reference range, 1.2–3.2)	Syrian	female	fetuses	deceased (20 weeks gestational age)	heart; liver;	cardiomyopathy	IUGR, oligo/anhydramnios, generalized edema, cardio/hepatomegaly, cortical hemorrhages, and preterm birth	Alstrup et al. (23)
11	c.118dupA; p.Thr40Asnfs ^*^45; c.612C>G; p.Phe204Leu	compound heterozygous mutations	1	truncation; coiled- coil region	NA	NA	NA	female	7 months	deceased (7 months)	heart	left ventricular hypertrophy, cardiac failure, ventricular arrhythmias	NA	Webster et al. (24)
12	c.118dupA; p.Thr40Asnfs ^*^45; c.612C>G; p.Phe204Leu	compound heterozygous mutations	1	truncation; coiled- coil region	NA	NA	NA	male	birth	deceased (27 days later)	heart	left ventricular hypertrophy, cardiac failure; ventricular arrhythmias	NA	Webster et al. (23)

*CNS, central nervous system; CS, citrate synthase; NA, not available; MRC, mitochondrial respiratory chain; PEO, progressive external ophthalmoplegia; PNS, peripheral nervous system; IUGR, intrauterine growth restriction*.

**Table 2:** The relative frequency of symptoms associated with biallelic variants in C1QBP and the related HPO terms.

**HPO terms**	**Frequency**	**C1QBP mutations**
Autosomal recessive inheritance	12/12	(p. Cys186Ser; p. Phe204Leu); (p. Gly247Trp; p. Leu275Pro); (p. Leu275Phe); (p. Tyr188del); (p. Phe204Leu); (p.Val248Ala); (p.Phe204Leu; p.Thr40Asnfs^*^45)
Cardiomyopathy	10/12	(p. Cys186Ser; p. Phe204Leu); (p. Gly247Trp; p. Leu275Pro); (p. Leu275Phe); (p. Tyr188del); (p.Val248Ala); (p.Phe204Leu; p.Thr40Asnfs^*^45)
Left ventricular hypertrophy	7/12	(p. Cys186Ser; p. Phe204Leu); (p. Leu275Phe); (p. Tyr188del) (p.Phe204Leu; p.Thr40Asnfs^*^45)
Ptosis	6/12	(p. Leu275Phe); (p. Tyr188del); (p. Phe204Leu)
Exercise tolerance	5/12	(p. Leu275Phe); (p. Tyr188del)
Progressive external ophthalmoplegia	5/12	(p. Leu275Phe); (p. Tyr188del); (p. Phe204Leu)
Cardiomegaly	4/12	(p. Cys186Ser; p. Phe204Leu); (p. Gly247Trp; p. Leu275Pro); (p.Val248Ala)
Hepatomegaly	3/12	(p. Gly247Trp; p. Leu275Pro); (p.Val248Ala)
Astigmatism	2/12	(p. Leu275Phe)
Generalized edema	2/12	(p.Val248Ala)
Ventricular arrhythmias	2/12	(p.Phe204Leu; p.Thr40Asnfs^*^45)
Amblyopia	1/12	(p. Leu275Phe)
Cardiorespiratory arrest	1/12	(p. Cys186Ser; p. Phe204Leu);
Cerebral edema	1/12	(p. Cys186Ser; p. Phe204Leu);
Dysphagia	1/12	(p. Phe204Leu)
Fatigue	1/12	(p. Leu275Phe)
Hypothyroidism	1/12	(p. Cys186Ser; p. Phe204Leu)
Nephrotic syndrome	1/12	(p. Cys186Ser; p. Phe204Leu);
Sensory neuropathy	1/12	(p. Leu275Phe)
Sensorineural hearing impairment	1/12	(p. Tyr188del)
Seizures	1/12	(p. Cys186Ser; p. Phe204Leu);
Vomiting	1/12	(p. Leu275Phe)

*HPO, Human Phenotype Ontology*.

*Variants in () refer to mutation. Two sites indicating compound heterozygous mutations, one site indicating homozygous mutations*.

The p.Phe204Leu mutation of the C1QBP protein was identified in four patients either in homozygosity (case 5) (21) or in compound heterozygosity (case 1, 11, 12) (20, 24). The homozygous p.Phe204Leu mutation described in case 5 showed an adult onset mild phenotype, with PEO and mitochondrial myopathy. The patient is alive without cardiac phenotypes (21). A further three patients with a p.Phe204Leu mutation were identified with a second mutation. The compound heterozygous p.Phe204Leu and p.Cys186Ser mutation occurred in case 1, a newborn baby with cardiorespiratory arrest, asymmetric left ventricular cardiomegaly, multiple cortical, ventricular, and subdural hemorrhages, cerebral edema and burst suppression-like electrical discharges with subclinical seizures (20). The heterozygous frameshift c.118dupA insertion can result in a truncation mutation, p.Thr40Asnfs^*^45, in the protein (23). The compound heterozygous p.Thr40Asnfs^*^45 and p.Phe204Leu mutations were noted in two siblings (case 11 and 12) with ventricular arrhythmias, cardiac hypertrophy and cardiac arrest.

The homozygous mutation of p.Leu275Phe was identified in cases 3, 7 and 8. These three patients are all alive and show clinical phenotypes of left ventricular hypertrophy, exercise intolerance and ptosis (20, 22). The homozygous p.Val248Ala mutations were described in case 9 and case 10 (23). They are associated with severe intrauterine growth restriction, edema, and cardiomyopathy. The compound heterozygous p.Gly247Trp and p.Leu275Pro mutations were identified in case 2 who suffered with cardiomegaly and hepatomegaly. She was deceased 4 days after birth. The homozygous p.Tyr188del mutation was identified in case 4 and was associated with left ventricular hypertrophy, exercise intolerance, weakness, ptosis, PEO and peripheral nervous system abnormalities (20). However, this mutation in Marchetet's report was identified with the phenomenon of PEO and mitochondrial myopathy, without cardiac involvement (21).

### Genotype-Phenotype Correlation

Among the 12 patients, cases 4, 5 and 6 showed later onsets and longer survivals compared with the remaining patients (20, 21). Their corresponding sites of amino acid change of p.Phe204Leu and p.Tyr188del all fell in the C1QBP protein coiled-coil region. The p.Cys186Ser, p.Gly247Trp, p.Val248Ala, p.Thr40Asnfs^*^45, p.Leu275Phe and p.Leu275Pro variants associated with early onset cardiomyopathy all occurred in important structural domains, such as the β strand, hydrogen bonded turn, and the αC helix. In particular, the p.Leu275Phe mutation is located in the αC helix, and three patients carrying this mutation all remain alive despite early onset combined OXPHOS deficiency and cardiac hypertrophy. These observations suggest that the localization of the pathogenic variants within the C1QBP protein structure show correlations with the various observed phenotypes.

### C1QBP Variant Protein Structure Predictions

The three-dimensional (3D) C1QBP structure was analyzed using the wild type protein (PDB accession codes 1P32, https://www.rcsb.org/structure/1P32) and the SWISS-MODEL (http://swissmodel.expasy.org/). Figure 1C was acquired with the PyMOL molecular graphics system (PyMOL, https://pymol.org/2/). The truncation mutation of p.Thr40Asnfs^*^45, located in the N-terminal mitochondrial targeting peptide proteolytically processed after C1QBP import into the mitochondrion, was not present in the 3D structure. Amino acid changes have pivotal effects on protein structure and hydrophobic surface exposure, especially the polarity and hydrophilic/hydrophobic differences after mutation. Cys186 and Ser186 are neutral amino acids. Val248 and Ala248 are nonpolar amino acids with hydrophobic side chains. Phe 204, Phe275 and Pro 275 have aromatic amino acid side chains, Leu204, Leu275 with fatty acid side chain, are all nonpolar. Thus the mutations of Cys186Ser, Val248Ala, Phe204Leu, Leu275Phe and Leu275Pro may not significantly alter the protein structure. Tyr is the aromatic amino acid with hydrophobic side chains; thus the Tyr188del mutation may result in a decreased exposure of the hydrophobic surface. Gly247 and Try247 have different polarity and side chains, suggesting that that the Gly247Tyr variant may increase the exposure of the hydrophobic surface. However, perspectives from structural change alone may not be sufficient to analyse the functional alterations without further verification.

### MtDNA Damage

The mtDNA damage is often associated with PEO (52). mtDNA copy number variants and multiple mtDNA deletions were identified in cases 1-8. Case 1 (p.Cys186Ser; p.Phe204Leu) and case 2 (p.Gly247Trp; p.Leu275Pro) showed higher mtDNA copy numbers in muscle and liver samples, but there was no evidence of mtDNA rearrangements (20). Case 3 (p.Leu275Phe) and case 4 (p.Tyr188del) showed multiple mtDNA deletions in muscle samples; both have a PEO phenotype (20). Similarly, Marchet et al. (21) reported two unrelated adult patients, presenting with PEO; muscle biopsies from both carried multiple mtDNA deletions. Our group reported a homozygous C1QBP-Leu275Phe mutation in case 7 and case 8 with ptosis instead of PEO, and no mtDNA deletion was detected in the blood samples of both patients and their parents (22). Nevertheless, determining whether PEO is linked to C1QBP mutation and mtDNA damage needs an expansion of the cohort of patients carrying C1QBP.

### Physiological Consequences

Muscle biopsies were obtained and described in cases 1–6 and cases 9 and 10. Feichtinger et al. (20) described four individuals, all with cardiac symptoms. Their respiratory chain activities in muscle or liver homogenates showed severe deficiency. Muscle homogenates from proband case 1, case 3, and case 4 showed decreased complex I and complex IV subunits, consistent with the findings of enzymatic investigations from muscle (20). Furthermore, case 3 and case 4 showed increased mitochondrial mass indices. Marchet et al. reported two unrelated adult patients from consanguineous families, presenting with PEO, mitochondrial myopathy, without cardiac involvement. Muscle biopsies from both patients showed the typical mitochondrial alterations. Spectrophotometric analysis of the mitochondrial respiratory chain complexes in muscle homogenates showed partially reduced complex I, III, and IV activities in case 5, whereas values were in the control range for case 6 (21). Alstrupet et al. reported that the analysis of a fibroblast culture from one of the fetuses showed a deficiency of respiratory chain complex IV (23). The muscle biopsy analysis indicates that the degree of respiratory chain complex deficiency may correlate with phenotype and genotype.

## Models of Mitochondrial Disease Associated With C1QBP Mutations

### Animal Model

Toshiro et al. generated cardiomyocyte-specific conditional C1QBP knockout (cKO) mice using the Cre-loxP approach (15). C1QBP-deficient mouse hearts showed altered mitochondrial structure and function corresponding to an increased oxidative stress, further leading to cardiomyocyte dysfunction. Furthermore, C1QBP-cKO mice presented with embryonic lethality with the embryonic fibroblasts showing multiple OXPHOS defects. Oxygen consumption rates, and mitochondrial and cytosolic translation were inhibited in C1QBP-cKO mice. C1QBP-deficient hearts also showed increased ornithine and decreased citrulline metabolites, suggesting that the urea cycle was affected. In conclusion, mitochondrial dysfunction caused by C1QBP deficiency affects cellular homeostasis and induces a protective response against cardiomyopathy (15).

### IPSC-CMs

Development of human induced pluripotent stem cells (hiPSC) has initiated a new era of *in vitro* cell model reconstruction and research into individual pathogenesis of mutation-specific diseases in patients. Somatic cells after reprogramming carry all genetic information including pathogenic genes. Cardiomyocytes differentiated from iPSCs (iPSC-CMs) can reproduce disease phenotypes, thus providing an important platform for studying pathogenesis (53). Our group has established iPSCs carrying the C1QBP-L275F mutation (54). The C1QBP-L275F-iPSC-CMs showed a cardiomyocyte hypertrophy phenotype in common with our patient. The cross-sectional area of iPSC-CMs derived from the proband was significantly increased compared to the mothers'. The C1QBP protein was distributed in the mitochondria. Electron microscopy showed that these were disordered in their morphology, number and size. Therefore, this is likely to become a successful model to provide a pivotal platform for studying the pathogenesis of mitochondrial cardiomyopathy caused by C1QBP-L275F mutations.

## Summary

We summarize the structure and physiological functions of C1QBP and its mutation related clinical phenotypes. C1QBP localizes predominantly in the mitochondrial matrix and is essential for OXPHOS maintenance. Patients identified with C1QBP mutations showed combined respiratory-chain deficiencies and abnormalities in the heart, liver, kidney, skeletal muscle, eye and nervous system. Clinical manifestations included intrauterine growth restriction, cardiomyopathy, hepatomegaly, exercise intolerance, swallowing dysfunction, ptosis, PEO and peripheral nervous system dysfunction. The relationship between observed mitochondrial cardiomyopathy and the C1QBP mutations and its underlying mechanism requires further studies for its elucidation on the platforms of iPSC-CMs and animal models.

## Author Contributions

YZ wrote the original manuscript. YZ and CL-HH reviewed and edited the manuscript. All authors contributed to the article and approved the submitted version.

## Funding

This work was supported by grants from National Natural Science Foundation of China (81974014 and 81470452); International cooperation project of Shaanxi Science and Technology Department (2019KW-072); Scientific research project of Xi'an Talent Program (XAYC200023) and Xi'an Children's Hospital Science Project (2020D01).

## Conflict of Interest

The authors declare that the research was conducted in the absence of any commercial or financial relationships that could be construed as a potential conflict of interest.

## Publisher's Note

All claims expressed in this article are solely those of the authors and do not necessarily represent those of their affiliated organizations, or those of the publisher, the editors and the reviewers. Any product that may be evaluated in this article, or claim that may be made by its manufacturer, is not guaranteed or endorsed by the publisher.
